# Can We CanMEDS? Intangible Learning Through Tangible Simulation Case Development

**DOI:** 10.7759/cureus.685

**Published:** 2016-07-13

**Authors:** Sabrina Alani, Holly Black, Chris Harty, Justin Murphy, Desmond Whalen, Kerry-Lynn Williams

**Affiliations:** 1 Emergency Medicine, Memorial University of Newfoundland; 2 Oncology, Memorial University of Newfoundland; 3 Faculty of Medicine, Memorial University of Newfoundland

**Keywords:** canmeds, medical simulation, medical education, continuous education

## Abstract

The Royal College CanMEDS framework has become a guide for medical school curricula. This framework aims to improve patient care by identifying and explaining seven key roles that physicians must fulfill in order to deliver high-quality healthcare to their patients. While medical schools incorporate these roles in their teaching processes, students can also apply them outside the classroom. Here, we describe a unique model developed at Memorial University of Newfoundland’s Tuckamore Simulation Research Collaborative (TSRC), where students develop simulation cases with the guidance of expert mentors and apply the Royal College CanMEDS framework to writing clinical simulations.

## Editorial

### Introduction

The CanMEDS framework aims to improve patient care by identifying and explaining seven key roles physicians must fulfill in order to deliver effective healthcare to their patients and communities [1]. Post- and undergraduate educational processes highlight these roles, but their real-world clinical application may not be clear to novice trainees, perhaps because traditional curricula must sometimes adopt passive teaching and evaluation methods. However, active learning by immersion in a topic and bringing it to fruition as a scholarly product offers a perspective that may not be captured purely by student reading.

Simulation training fills some of the gaps between medical reading and medical practice. Writing our own simulation cases offers students an even better understanding. In this editorial, we describe a novel idea: undergraduate and graduate medical students develop simulation cases alongside experienced faculty members. Developing the simulation cases, which may be based on real or fictional patient encounters, adds realism that supplements our undergraduate education, and offers an opportunity for self-directed learning. It allows the medical trainee to pursue areas of interest with a depth that may not be possible within an already full traditional curriculum. Writing simulation cases helps us think like physicians and encourages us to refine our approach to patient management. It also helps us develop the CanMEDS roles. It brings the CanMEDS roles to life.

### What did we do, and how did we do it?

CanMEDS roles are an excellent framework for emerging physicians, but they needed a vehicle to deliver their principles to students. For us, that vehicle was mentorship from an interdisciplinary group of researchers, physicians, and medical and graduate students who collaborate in simulation-based health professions research.

This group introduced us to a collaborative writing process. Simulation helps identify the patterns we use when performing a medical procedure; writing about the experience reinforces those patterns. To date, members of this team have collaboratively written eight simulation cases and published them as technical reports in Cureus. This exercise has given medical students the freedom and confidence to pursue complex simulation cases. The collaboration between mentor and mentee becomes a transformative vehicle that turns novel ideas into tangible products worthy of publication. Interestingly, our simulation case writing activity allows us to master all seven CanMEDS roles at once.

### The CanMEDS roles

Scholar

The Scholar role mandates physicians to commit to ongoing teaching and learning. The scholar critically evaluates new information and applies it to practice, thus, facilitating the healthcare-related learning of patients and their community and contributing to new medical research and its dissemination.

Writing Cureus simulation cases fulfills this role. Every technical report provides an opportunity to conduct a literature review on a specialized topic. In our cases, for example, we researched diverse topics, such as ectopic pregnancy and burn management [2-3]. This approach taught us to complete literature reviews, critically appraise the literature, and understand clinical guidelines. Publishing these articles also allowed us to disseminate knowledge. By collaborating, we are able to integrate into practice best available evidence and valuable clinical pearls.

Leader

The Leader role requires physicians to contribute to a high-quality healthcare system by focusing on patient safety, leading change, balancing their career, practice, and personal life, and taking on various roles (manager, negotiator, teacher, etc.), all in order to achieve a sustainable healthcare practice and system.

Writing Cureus simulation cases allowed us to develop our leadership skills in many ways. First, students must lead in order to complete and publish a case. Second, the process taught us valuable lessons about time and resource management. Part of resource management is seeing your limitations, recognizing what you do not know, and knowing when to ask for clarification. Some of the cases required us to think hard about how a leader would act in certain circumstances. For example, as an emergency physician, one must remember that visitors in the emergency department might sometimes be privy to information they should not hear. Physicians must be prepared to manage these scenarios. Our pregnancy and privacy case used a real-life scenario and helped us, as student writers, to consider the importance of the context surrounding clinical management, a key character trait to develop as future physician leaders [2].

Collaborator

The Collaborator role requires the physician to work within a health care team for optimal patient care.

Just as a practicing doctor must work with other health care professionals to form a patient’s care plan, so too does an interdisciplinary Cureus team collaborate in writing an article. Writing a paper always begins with divisions of labor, followed by regular group discussions. As it is in the medical world, interdisciplinary team members need to come together to write and publish a case. Each member has a unique and valuable perspective. In writing cases, we consulted the medical content experts and senior researchers on our writing teams, just as we would the practicing physicians in an actual medical case.

Many cases involved interdisciplinary collaboration. For example, the Ebola virus article was led by nursing experts who noticed improperly donned protective equipment when treating potential Ebola patients [4]. This particular case brought forward different perspectives (nursing and research) and required us to merge our different perspectives and styles. Another case involving rural medevac and the use of a helicopter simulator [5] required us to collaborate with experts in prehospital and rural medicine, as well as engineering and aviation.

Collaborative writing also requires us to master negotiation skills by jointly discussing subjects like deadlines, role division, and the writing process. Making sure everyone is on the same page, troubleshooting unmet deadlines, or unexpected delays are all analogous to what a physician would do when collaboratively managing a real-life case with other health care professionals. Our teams were respectful, promoted a collaborative culture, and provided opportunities to build on each other’s strengths for the next time around. As is often the case with real-life medical scenarios, team members shared expertise and knowledge with each other. Knowledge gaps perceived by one team member were respectfully filled by another, all in order to achieve a common goal.

Communicator

The Communicator role requires the physician to facilitate the doctor-patient encounter, to synthesize and communicate both oral and written medical information, and to communicate information to patients and other health care professionals. This role focuses heavily on a physician’s ability to communicate with both patients and their caregivers.

Attending medical school is like learning a different language, in this case, full of acronyms and Latin. Writing these cases helped us achieve fluency. It familiarized us with general medical terms and their associated shorthand. It helped us learn how to record patient history and physical exam findings properly and efficiently. This is important clinically when students must succinctly communicate pertinent positives and negatives to staff physicians.

We are currently writing a series of pediatric cases, one of which emphasizes communication with the child’s parent. Another case focuses on the importance of collateral history in assessing a confused elderly patient. While the content of these cases all illustrate clinical communication, the act of writing them also requires that students communicate well with their team members, just as they will in future with healthcare professionals, patients, and their families.

Professional

The role of the Professional demands that physicians demonstrate a commitment to their patients and their practice, ethically and while adhering to regulations.

New medical learners are often exposed to a great deal of medical expertise that may overshadow the art of medicine. Understanding patient vulnerabilities may take second place to the treatment of disease. Writing these cases reminds us of the balance we will be expected to strike as practicing physicians. The pregnancy and privacy case, which required thinking not only about clinical findings but also about confidentiality, illustrates the point [2]. Cases like these help us to focus on both the art and the science of medicine and remind us that professionalism is key. Early exposure to these principles will help us as we go through our training and practicums and, later, our careers.

Health Advocate

As Health Advocates, physicians are expected to promote wellness for their patients and their communities.

Patients want and expect their health care providers to have information about their condition, about prevention, and about treatment plans. By writing these cases, we encourage this knowledge acquisition for both patients and their health care providers. These cases help improve clinical practice and allow us as student writers to look for and address information gaps and potential pitfalls.

Medical Expert

Finally, the role of Medical Expert is all-encompassing, the summation of all of the roles described above. As Medical Experts, physicians must be effective consultants, able to assess patients, prescribe therapeutic and diagnostic interventions, and seek expertise from other health professionals when necessary.

From the student perspective, writing the burn management case allowed us as writers to understand the flow of burn management in ways we did not before; for instance, knowing to look for signs of carbon monoxide poisoning and smoke inhalation in addition to treating visible burns [3]. Writing the scenario templates within Cureus requires us to consult with expert clinicians, in order to use best practices. Developing the case itself sharpens our clinical skills, bringing our attention to details like vital signs and other red flags that may portend clinical deterioration. All of this helps prepare us for our roles as medical experts.

### Conclusion

The development of the above-mentioned cases has strengthened our research skills and at the same time enabled application of the CanMEDS roles in ways not traditionally taught in the classroom. To date, we have established a rich repository of clinical simulation scenarios. We have developed cases from propranolol overdose to infant trauma management, a new and innovate mnemonic (SPIRAL) for emergency medicine trainees, and complex medical transportation and evacuation [5-8]. We have written about privacy in the emergency room, and proper logistical management during an Ebola outbreak [2, 4]. These cases have helped us gain valuable research skills, communicate and collaborate with fellow researchers and mentors, manage and lead a case from beginning to end, communicate results, and ultimately, gain medical knowledge.

We are confident that our work has been successful. To date, we have established a rich repository of clinical simulation scenarios. We have developed cases from propranolol overdose [6] to infant trauma management [8], from a new and innovate mnemonic (SPIRAL) for emergency medicine trainees [7] to complex medical transportation and evacuation [5]. We have written about privacy in the emergency room [2], and proper logistical management during an Ebola outbreak [4]. These cases have helped us gain valuable research skills, communicate and collaborate with fellow researchers and mentors, manage and lead a case from beginning to end, communicate results, and ultimately gain medical knowledge.

Our repository of simulation cases continues to expand. New medical students have become involved, while current students continue to find new and innovative means of developing simulation cases. Illustrating the interdisciplinary nature of our work, the helicopter emergency evacuation case brought together medical experts at the Faculty of Medicine, academics at the Marine Institute, and members of technology sector (Virtual Marine Technology) [5]. By collaborating in teams, we hope to create a community of practice that will benefit learners, professionals, and ultimately, our patients and their communities.
